# Betaine Contribution to Performance Enhancement in Sport

**DOI:** 10.3390/nu18203317

**Published:** 2026-10-10

**Authors:** Corina Flangea, Cristian Sebastian Vlad, Tania Vlad, Cristina Doriana Marina, Victor Dumitrascu, Bogdan Balasoiu, Daliborca Cristina Vlad

**Affiliations:** 1 Department of Biochemistry and Pharmacology, Faculty of Medicine, “Victor Babeș” University of Medicine and Pharmacy, Eftimie Murgu Sq. No. 2, 300041 Timisoara, Romania; flangea.corina@umft.ro (C.F.); cristina.marina@umft.ro (C.D.M.); dumitrascu.victor@umft.ro (V.D.); bogdan.balasoiu@student.umft.ro (B.B.); vlad.daliborca@umft.ro (D.C.V.); 2 Doctoral School, “Victor Babeș” University of Medicine and Pharmacy, Eftimie Murgu Sq. No. 2, 300041 Timisoara, Romania; tania.vlad@umft.ro; 3 ANAPATMOL Research Center, Department of Cell and Molecular Biology, Faculty of Medicine, “Victor Babeș” University of Medicine and Pharmacy, Eftimie Murgu Sq. No. 2, 300041 Timișoara, Romania

**Keywords:** betaine, homocysteine, athletic performance, sport nutrition, post-exercise recovery

## Abstract

Betaine is a compound with significant implications in exercise biology due to its ability to optimize metabolic homeostasis and exercise capacity. This narrative review analyzes the physiological and biochemical mechanisms by which betaine influences neuromuscular performance and regulates post-exercise recovery processes. From an ergogenic perspective, betaine acts as an organic osmolyte and a methyl group donor in the methionine resynthesis cycle, a process directly associated with improving muscle strength and anaerobic power. Its anti-inflammatory properties are mediated by suppressing the nuclear factor-kB (NF-kB) proinflammatory axis and IL-1β activity, thereby attenuating exercise-induced muscle damage. Metabolically, the compound exerts anti-adipogenic effects by downregulating key enzymes involved in lipogenesis and stimulating fatty acid β-oxidation, which favors the optimization of body composition. Furthermore, its vasodilatory action is primarily attributed to the potentiation of the endothelial nitric oxide synthase (eNOS) pathway, ensuring optimal perfusion and nutrient delivery to working muscles. At the central level, the neuroprotective effect consists of counteracting neuronal oxidative stress, clearing homocysteine and attenuating antidepressant behavior. These data reveal that betaine is a complex plurivalent ergogenic agent. Its integration into sports nutrition optimizes the adaptive physiological response to exercise through integrated cellular, vascular and metabolic mechanisms, representing a viable strategy for the overall improvement of athletic performance.

## 1. Introduction

Dietary supplement intake has a long history of use in the world of professional athletes and is also widespread in the general population. According to the International Olympic Committee, dietary supplements can be perceived as a food, food component, non-food product, or nutrient consumed deliberately with the aim of improving performance [1]. A wide range of sports supplements are already known worldwide, such as creatine, beta-alanine, nitrates, proteins and caffeine [2]. Regarding sports supplements, although they may enhance athletic performance, concerns have been raised about their potential adulteration with undeclared pharmacologically active substances, including anabolic steroids and prohormones [3]. Among the various types of supplements, there is currently growing interest in the use of betaine. Unlike traditional sport supplements such as creatine, beta-alanine and carnitine, betaine operates at the crossroads of physical cell protection and biochemical transformations. Thus, betaine maintains intracellular hydration, reduces plasma homocysteine levels, and accelerates protein synthesis. Furthermore, it can epigenetically modulate DNA methylation patterns and increase the endogenous synthesis of creatine and carnitine [4,5,6]. Consequently, its comprehensive biological impact cannot be replicated by a single-ergogenic-effect point of view targeting evaluation of its performance-enhancing and recovery potential following sport-related trauma for athletes. Increasingly utilized as an ergogenic supplement, betaine is a trimethylated derivative of glycine that donates methyl groups to homocysteine, supporting the formation of S-adenosylmethionine [7]. Thus, betaine can be synthesized in vivo through choline metabolism and can also be obtained through dietary intake [7]. It is known as a well-tolerated substance found abundantly in microorganisms, animals, and plants [8,9]. Over the years, research has demonstrated that natural sources of betaine include beets, spinach, bran, wheat germ, and seafood [8,9]. Betaine is absorbed in the small intestine and distributed to the tissues, where it plays an important role in metabolic processes and osmotic hemostasis [10]. This natural compound is attracting interest in the pharmaceutical industry and the dietary supplement sector due to its low toxicity and promising post-injury recovery properties [11]. A particularly important consideration concerns the difference between betaine intake from dietary sources compared to supplementation. Given the variability of dietary patterns, supplementation is sometimes necessary to achieve optimal levels, which are considered to be around 30 µmol/L for healthy subjects [12] and approximately 50 µmol/L post-supplementation in athletes [13], with individual variations ranging from 9 to 90 µmol/L [12,13]. This situation is frequently encountered among elite athletes who engage in intense physical exertion. A study conducted on university athletes showed that supplementation with 5 g/day of betaine for six weeks may contribute to improved muscle strength [14]. Other studies reported in the scientific literature have shown that betaine, when included in resistance training programs, can improve body composition and performance [15]. Although there are already review articles on this topic in the scientific literature, these are generally based on the beneficial potential, which is why more investigations should be performed [15,16,17,18]. Recently, Perreras and Kim published a systematic review of betaine supplementation for endurance enhancement, their study once again highlighting the lack of a considerable body of evidence regarding its ergogenic potential [19]. A randomized trial conducted for 6 weeks with betaine 2.5 mg per day found no notable results, suggesting that many factors, such as dose, duration of intervention, and lifestyle, may play a significant role [20]. Moreover, a study published in 2021 showed that although betaine has promising effects, more research is needed to examine changes in physical performance indicators [21]. Therefore, while betaine has begun to be seriously researched, more studies and analyses are needed to show us how betaine supplementation can increase athletic performance from moderate to high level. In addition to the ergogenic effect observed in athletes, betaine has also begun to be studied in other contexts. Research has shown that it holds potential for alcohol-induced hepatic steatosis; furthermore, lower betaine concentrations have been associated with greater insulin resistance [22,23]. Future investigations will likely lead to a better understanding of the properties of betaine and indicate its relevance in complex pathologies.

In today’s competitive sports, the physical demands of prestigious competitions are consistently rising, putting immense pressure on high-performance athletes. Consequently, athletes seek optimal dietary supplements or nutrition strategies that can genuinely help them improve performance, enhance post-exercise recovery, and prevent or accelerate the healing from injuries. In this narrative review, we highlight the specific properties of betaine that could enhance athletic performance, focusing not only on the primary mechanisms underlying these physical improvements, but also integrating disparate biological pathways and synthesizing current evidence regarding its efficacy in athletes to foster discussions that may pave the way for future research. Unlike previous studies that focus strictly on conventional exercise performance or isolated aspects of betaine, our paper provides an up-to-date critical analysis of its integrated role from multiple perspectives. By examining existing findings and evaluating conflicting data, this work defines the practical application of betaine supplementation—an analysis uniquely conducted from the dual perspective of a medical researcher and a former elite athlete.

## 2. Methodology

The aim of this narrative review is to discuss and analyze current controversies regarding betaine’s ability to enhance athletic performance. While systematic reviews are highly effective for analyzing uniform clinical trials, a narrative review is uniquely suited for mapping the current landscape of betaine research. This approach allowed us to address the complex biochemical, metabolic, and physiological transformations associated with betaine supplementation in sports. Furthermore, a narrative review enables an integrated perspective—synthesizing diverse viewpoints and contextualizing specific findings—while also highlighting potential directions for future research. To achieve this integrated perspective, the review begins by describing the metabolic processes involving both endogenous and exogenous sources of betaine establishing a foundation for understanding its ergogenic potential. The section dedicated to the mechanisms potentially influencing an athlete’s ability to achieve peak performance presents current knowledge on this controversial topic. In addition to its direct ergogenic properties, we also address other indirect yet significant factors contributing to the overall impact: anti-inflammatory, anti-adipogenic, vasodilatory, and neuroprotective effects. Although some studies utilize pure betaine, results obtained from beetroot juice—one of the most important natural sources of betaine—cannot be overlooked. Consequently, we considered findings from its administration, acknowledging that it is a complex mixture where other components might influence specific physiological and biochemical responses. The final section is dedicated to discussions and hypotheses that could guide future research, helping to answer outstanding questions or pave the way for new investigations.

To address this objective, we used various keywords—such as “betaine,” “sports performance,” “betaine synthesis,” “betaine absorption,” “betaine distribution,” “betaine metabolism,” “betaine elimination,” “ergogenic effect of betaine,” “daily betaine dosage,” “betaine–testosterone relationship,” “methyl donors,” “betaine anti-inflammatory,” “betaine adipose tissue,” “beetroot and beetroot juice,” “homocysteine,” “betaine vasodilator,” “NMDA betaine homocysteine,” and “betaine/beetroot juice neuroprotection”—along with various combinations thereof. These keywords were used to search databases including PubMed, ScienceDirect, Google Scholar, and Web of Science. Selected articles were limited to peer-reviewed original research, reviews, and case report, whereas meeting abstracts, letters to the editor, editorials, commentaries, and conference proceedings were excluded. WADA documents were also consulted. The search strategy was flexible, taking into account mechanistic descriptions and other details found in introductory sections, as well as information providing further explanation of biological processes.

## 3. Betaine Physiological Synthesis in the Human Body and Related Conversions

In humans, betaine plasma concentrations (20–70 µM/L) are maintained through a combination of endogenous synthesis and dietary intake [9]. Synthesis occurs in the mitochondria of the liver and kidneys via two irreversible steps: first, the oxidation of choline to betaine aldehyde, mediated by choline dehydrogenase and coenzyme Q; and second, the subsequent oxidation of betaine aldehyde to betaine, catalyzed by betaine aldehyde dehydrogenase. Following mitochondrial synthesis, betaine is transported into the cytosol to serve as a methyl donor in the remethylation of homocysteine to methionine [9,24,25]. It is in generally estimated that approximately 40% of choline is converted to betaine [26]. The conversion of homocysteine to methionine is a complex process facilitated by two distinct pathways: the betaine-dependent pathway, which converts betaine to N,N-dimethylglycine via betaine homocysteine methyltransferase, and the folate-dependent pathway, which utilizes methionine synthase to convert 5-methyltetrahydrofolate to tetrahydrofolate. The reverse transformation of methionine to homocysteine occurs indirectly. It begins with the addition of an adenosyl group to form S-adenosylmethionine, a reaction catalyzed by methionine adenosyltransferase. S-adenosylmethionine is then converted to S-adenosylhomocysteine by S-adenosylmethionine-dependent methyltransferases. Finally, S-adenosylhomocysteine is reversibly hydrolyzed to homocysteine by the enzyme S-adenosylhomocysteine hydrolase [24,27,28].

From this point, betaine indirectly supports the production of endogenous taurine through a series of steps involving the formation of cystathionine and cysteine. First, cystathionine β-synthase acts on homocysteine to form cystathionine, which is then converted into cysteine. Subsequently, cysteine dioxygenase facilitates the transformation of cysteine into taurine [24]. All of these transformations are illustrated in Figure 1.

Even though endogenous synthesis occurs, it is generally considered insufficient to meet physiological demands, especially in athletes [9]. In this context of increased nutrient requirements, exogenous intake—from both food sources and dietary supplements—becomes beneficial.

## 4. Pharmacokinetics of Exogenous Betaine

Betaine is a natural occurring compound found in various sources such as microorganisms, wheat, spinach, and fruit peel. The most significant source is beetroot (3980 µg/g), though concentrations vary by species: golden *Beta vulgaris* (4330–5070 µg/g) and red *Beta vulgaris* (1960–3880 µg/g) [29]. Notably, betaine remains stable during extraction [30], as it is characterized by high chemical stability [31].

### 4.1. Absorption and Distribution

Exogenous dietary intake varies significantly, averaging between 1 and 2.5 g/day [32]; however, broad ranges have been reported, spanning from 0.1–0.4 g/day [20,33] to as much as 9–12 g/day [34]. Betaine is absorbed in the intestine via both sodium-dependent amino acid transport system A and sodium-independent pathways [33,34]. Under hypertonic stress, betaine is also absorbed through membrane-bound betaine/gamma-aminobutyric acid transporter 1 [32]. Peak distribution occurs approximately 1–2 h following oral administration and intestinal absorption [9]. In diets deficient in methyl donors, a reduction in methionine and betaine is observed, alongside a more than 100-fold increase in accumulation of homocysteine and S-adenosylhomocysteine [35].

Betaine from food and dietary supplements exhibits similar bioavailability, although absorption is 33% higher when taken as a supplement [36]. It accumulates in the body as an organic osmolyte, providing a cytoprotective effect against cellular hypertonicity during water stress [34,37] and contributing to heat stress tolerance [33].

Consequently, betaine is a stable compound that does not undergo transformations under standard environmental conditions. Furthermore, its high bioavailability and osmolytic capacity during heat stress make it valuable when physiological demands increase. It is therefore evident that in sports, where prolonged physical effort increases nutrient demands, betaine supplementation can be advantageous.

### 4.2. Metabolism and Elimination

During the conversion of homocysteine to methionine, betaine irreversibly loses a methyl group and is converted to N,N-dimethylglycine in a reaction catalyzed by betaine–homocysteine methyltransferase [24,27]. The resulting metabolite is then eliminated renally [38,39]. N,N-dimethylglycine can be further converted to sarcosine via mitochondrial demethylation, a process mediated by the enzyme dimethylglycine dehydrogenase. Sarcosine is subsequently converted to glycine in a reaction catalyzed by sarcosine dehydrogenase [24,40,41] (Figure 2). In the liver, betaine can be metabolized to trimethylamine by betaine reductase and then oxidized to trimethylamine N-oxide (TMAO) by trimethylamine monooxygenase. The resulting TMAO is not further metabolized and is eliminated renally [42].

Betaine is known to have a very low median clearance (approximately 2 mL/min/1.73 m^2^) and can be effectively removed during a single hemodialysis session [43]. Although its half-life is estimated at 14.38 h [9], Machek S.B et al. demonstrated that following a 14-day supplementation period of 6 g/day in five endurance athletes, plasma concentrations returned to baseline after a subsequent 14-day washout period [44].

The decline in plasma concentration shortly after the cessation of supplementation—even with intact liver and renal function—suggests that continuous may be necessary to maintain ergogenic benefits, particularly for the endurance athletes mentioned above [44]. Additionally, heat stress during intense muscular activity may be a significant contributing factor. Conversely, an alternative hypothesis suggests that the body excretes any surplus it does not require, rendering supplementation ineffective. Exploring these competing hypotheses in further research would be a valuable strategy to determine which more accurately reflects the physiological reality.

## 5. Mechanisms Involved in Performance Improvement in Sports

Betaine is common ingredient in dietary supplements and is often consumed in supraphysiological amounts, such as when performance athletes incorporate a whole beetroot into natural shakes between meals. Within this context, this section explores the primary mechanisms and physiological explanation for betaine’s role in enhancing athletic performance.

### 5.1. Ergogenic Effect

The ergogenic effect of betaine as a methyl donor is attributed to enhanced creatine and carnitine synthesis, which maintains water homeostasis [41,45]. In soccer players, a single 70 mL dose of commercially available beetroot juice concentrate improved change-of-direction performance and post-sprint agility [46]. Thus, acute administration of beetroot juice is suggested to increase peak sprint power without affecting endurance [47,48]. Chronic supplementation at doses of 1.25–2.5 g/day has been shown to delay the onset of muscle fatigue during endurance activities [41]. Furthermore, long-term administration of supplements containing 2.5 g of betaine consistently demonstrates ergogenic properties [49]. In a study conducted on college athletes, supplementation with a 5 g/day of betaine for 6 weeks demonstrated significant improvements in sports performance compared to placebo; however, no changes in lipid profile were identified [14]. In female athletes, betaine supplementation at a dose of 2.4 g/day for 2 weeks was shown to improve sprint performance, particularly during the final stage of the sprint. Another notable finding was a concomitant increase in fat free mass [50]. This may be attributed to enhanced muscle lactate clearance and the preferential utilization of fatty acids as an energy substrate [51]. Furthermore, betaine acts as an osmolyte maintaining cellular hydration during intense muscular exertion compounded by dehydration and hyperthermia. By preserving intracellular volume, betaine exerts a protective effect on Krebs cycle enzymes, allowing them to maintain high catalytic activity even under thermal, hydric and metabolic stress [51,52]. Table 1 provides a summary of randomized controlled trials evaluating the impact of betaine supplementation in various sports disciplines.

Another hypothesis is based on a reported 7% increase in plasma testosterone concentration following 2.5 g/day supplementation alongside performance enhancement in CrossFit athletes, specifically an increased number of repetitions across all three rounds of the “Fight Gone Bad” workout [56]. This hypothesis is further supported by a documented rise in testosterone/cortisol ratio after professional soccer players received 2 g/day of betaine for 14 weeks. Additionally, this intervention led to a reduction in chronic inflammation, accompanied by improvements in athletic performance metrics, including the 30 m sprint, vertical jump and global body strength [57]. Betaine can furthermore increase growth hormone (GH) and insulin-like growth factor-1 (IGF-1) levels independently of lactate production. In this context, the combination of betaine and blood flow restriction may act synergistically via the enhancement of hypoxia-inducible factor (HIF)-1A gene expression to augment anabolic potential [54]. Furthermore, betaine has been shown to increase hemoglobin levels, hematocrit, and red blood cell count [34,54], potentially through stimulation of testosterone production.

Recent data have demonstrated that betaine mimics physical activity in the body by activating AMP-activated protein kinase (AMPK), reducing oxidative stress, and improving mitochondrial function. These effects have led to exploration of its role in chronic diseases [8]. Furthermore, a high dietary intake of methyl donor compounds, including betaine, protects against metabolic alterations in obese and overweight adolescents [58].

Despite the abovementioned evidence, several studies failed to demonstrate any increase in muscle strength and power following betaine supplementation, including among CrossFit athletes [19,20,45,59]. Another study demonstrated modest improvements in cyclists during a 60 km time trial following the administration of 3 g/day of betaine for two weeks [7]. While several clinical trials indicate that betaine supplementation can improve certain physical aspects across various sports, these ergogenic effects are not consistently demonstrated. These discrepancies in findings may be attributed to the training status, the specific sport practiced, and individual metabolic characteristics.

### 5.2. Anti-Inflammatory Effect

The anti-inflammatory effects of betaine may contribute to enhanced athletic performance during elite competitions and subsequent recovery periods, where minor trauma—accompanied by acute inflammation and pain—can negatively affect outcomes.

This anti-inflammatory potential has been demonstrated through the inhibition of toll-like receptor-4 (TLR4) hypermethylation in skeletal muscle [24,60], the suppression of nuclear factor-kB (NF-kB) and IL-1β activities, and the prevention of leucine-rich pyrin-containing-3 (NLRP3) inflammasome activation [24,61]. In a study involving 29 youth soccer players evaluated during the competitive season, 14 subjects received betaine at a dosage of 2 g/day, while the remaining participants served as the placebo group. After 14 weeks, a significant reduction was observed in the plasma concentrations of IL-1β, IL-6, and TNFα associated with the exercise-induced stress of soccer matches [21]. In mice with induced osteoarthritis, betaine supplementation was observed to reduce the loss of articular proteoglycans, as evidenced by a decreased number of cells expressing matrix metalloproteinase 13 (MMP13) and collagen X [62]. Despite these localized protective observations, Xu J et al. reported no significant reduction in the systemic concentrations of IL-1β, CRP and TNFα [63].

### 5.3. Anti-Adipogenic Effect

In sports, reducing adipose tissue mass is a frequent concern. Although objectives vary—ranging from meeting strict weight categories in combat sports to minimizing additional ballast that impairs biomechanical execution in speed and pair sports—there is a universal desire to optimize body composition by reducing body fat.

The role of betaine in modulating and reducing adipose tissue has been highlighted in several studies seeking to explain this effect. In mice fed a high-fat diet (HFD) and supplemented with betaine, a reduction in body weight was observed compared to the control group. Betaine has been shown to stimulate lipolysis, inhibit lipogenesis in adipose tissue and improve mitochondrial function. This was demonstrated by an enhancement of fatty-acid oxidation, associated with the increased expression of β-oxidation genes, such as carnitine palmitoyl transferase 1A (CPT1A), as well as the mitochondrial peroxisome proliferator-activated receptor γ coactivator 1α (PGC 1α) [64]. In addition to the translocation of free fatty acids (FFAs) into mitochondria via CPT1A, the downregulation of enzymes involved in lipogenesis is evident, including acetyl-CoA carboxylase (ACC), malic enzyme (ME), fatty acid synthase (FAS) and sterol regulatory element-binding protein-1c (SREBP-1c) [4] (Figure 3). Furthermore, betaine can induce the browning of white adipose tissue [4]. In pigs, betaine can also reduce FAS activity and increase hormone-sensitive lipase activity in adipose tissue [65,66,67]. Additionally, it can reduce ACC activity by modulating its phosphorylation through the regulation of AMPK [22]. Furthermore, the conjugation of betaine with linoleic acid can enhance lipolysis and reduce adipose tissue mass by decreasing glycerol release [65]. At the hepatic level, betaine can help alleviate hepatic steatosis, including alcohol-induced steatosis, by reducing triacylglycerol (TAG) accumulation [22]. Under HFD conditions, betaine can increase short-chain fatty acid production, which subsequently enhances fatty acid oxidation and thermogenesis in adipose tissue, while simultaneously improving mitochondrial function. Consequently, betaine has been observed to reduce obesity associated with metabolic disorders, particularly under conditions of increased insulin resistance. This effect was observed in SD rats fed a HFD and supplemented with 1.5% betaine in their drinking water daily for eight weeks. In these rats, betaine supplementation also promoted the proliferation of beneficial microbiota and the generation of short-chain fatty acids [68]. Consequently, betaine appeared to protect the intestinal barrier both by modulating the beneficial microbiota and by upregulation tight junction proteins, specifically occludin, claudin-1 and zonula occludens-1 [32]. Thus, in mice and rats fed a HFD, betaine has also been shown to reduce circulating FFAs, thereby improving hypertriglyceridemia [67]. The excess circulating glycerol resulting from this supplementation can participate in gluconeogenesis or be oxidized through conversion to glycerol 3-phosphate (G3P), ultimately generating energy in the form of ATP [69] (Figure 3).

Betaine intervention and endogenous synthesis depend on choline availability. Regarding the expression of SLC25A48—a transporter located in the inner mitochondrial membrane that is upregulated under HFD conditions and required for the intramitochondrial transport of choline and subsequent betaine synthesis—it has been observed that choline uptake enhances betaine synthesis via SLC25A48, thereby protecting against oxidative stress. In the case of brown adipose tissue-derived cells lacking SLC25A48 (isolated from SLC25A48-KO mice), exogenous betaine supplementation was insufficient to protect cells against reactive oxygen species (ROS) [70]. Furthermore, Sivanesan S et al. suggested that the concomitant administration of choline and betaine can induce a state that mimics fasting [69]. Beyond choline’s role as a metabolic precursor of betaine, the concomitant administration of both compounds may enhance lipolysis, thereby facilitating weight loss [69,71]. Moreover, this effect of betaine appears to be synergistic with that of curcumin [72], which supports the hypothesis of evaluating this combination in dietary supplements designed for weight loss. In this combination, curcumin induces the browning of adipose tissue and reduces body mass index and waist circumference [73,74], while betaine—in addition to the anti-adipogenic effect discussed above—can prevent the loss of lean mass by protecting cell membrane integrity, acting as an osmolyte and methyl donor [33,34,37,58]. From a pharmacokinetic perspective, betaine has a low potential as candidate for enhancing curcumin bioavailability. In practice, there are significant pharmacokinetic limitations: betaine is a highly hydrophilic molecule with rapid absorption, whereas curcumin is a hydrophobic substance with low bioavailability. Although there is a trend toward combining the two molecules, no controlled human trials have been conducted yet. Nevertheless, a study has recently investigated the potential of betaine to serve as a carrier for curcumin uptake [75]. In this context, further research is warranted to elucidate their potential synergistic mechanism in metabolic contexts.

### 5.4. Vasodilation Effect

Vasodilation in sport is crucial for ensuring blood and oxygen supply to skeletal muscle and the heart. This aspect becomes more complex when betaine is ingested in the form of beetroot juice/powder, given its high concentration in inorganic nitrates. Bentley RF et al. [76] observed that beetroot juice supplementation induces compensatory vasodilation and improves exercise capacity by restoring muscle perfusion in individuals with reduced exercise tolerance. Several hypotheses could explain these findings, the first being the generation of a NO surplus.

During exercise, with an increase in heart rate, there is a release of NO, which plays a role in vasodilation. In general, moderate-intensity exercise has been shown to improve NO-mediated endothelial function and reduce oxidative stress, while high-intensity exercise increases oxidative stress [77]. Beetroot juice has been shown to induce NO release, improve endothelial function, and enhance athletic performance under conditions of prolonged muscular effort [78,79]. Modification of the NO_3_-NO_2_-NO pathway and modulation of endothelial nitric oxide synthase (eNOS) via dietary beetroot juice supplementation improves endothelial function, response to transient ischemia, and vascular cognitive impairment [80,81]. However, beetroot juice supplementation in individuals undergoing moderate-intensity training does not alter the cardiovascular response to such activities. This could be explained by the fact that lower-intensity exertion does not represent a strong enough vasodilatory stimulus to induce NO release [82]. Importantly, beetroot juice is naturally rich in NO_3_. Consequently, microencapsulation of beetroot powder may further amplify this effect [83]. Administration of betaine (10 mg/kg body weight) to spontaneously hypertensive rats (SHRs) and Wistar Kyoto rats (WKRs) for 7–8 weeks reduced blood pressure and decreased angiotensin-converting enzyme activity. In this study, the elevated plasma NO concentration was attributed to enhanced NO bioavailability following betaine administration, with the maximum effect observed in SHR [84]. Betaine has been shown to attenuate microvascular endothelial damage via the eNOS/NO pathway [85]. Although these animal models provide a mechanistic view as fundamental evidence regarding cellular protection and understanding how these findings could provide information on human active population. Additionally, plasma NO levels rose after a single dose of beetroot juice in both hemodialysis patients and healthy volunteers [86]. When transitioning to athletic cohorts, these observed physiological adaptations offer valuable insights into performance optimization. In amateur triathletes, beetroot-based nitrate supplementation has been shown to improve endurance and recovery capacity. This enhancement in athletic performance is driven by sustained NO-induced vasodilation and increased NO bioavailability, which subsequently optimize muscle perfusion and oxygen delivery during exercise [87]. Interestingly, beetroot juice supplementation in bodybuilders using anabolic steroids improved endothelial function but failed to elicit other cardiovascular benefits effects or enhance muscle performance [88]. To highlight these properties with ergogenic potential, research has also focused on active populations experiencing physiological decline. A 12-week follow-up of nitrate-rich beetroot juice supplementation in postmenopausal women—a population where endogenous NO production decreases alongside declining estrogen levels—improved muscle strength and contractile properties [89]. Furthermore, this intervention enhanced post-exercise recovery within this cohort [90].

According to the second hypothesis, vasodilation may be indirectly driven by a reduction in homocysteine, a known vasoconstrictor associated with endothelial dysfunction [82,84]. Consistently, betaine supplementation reduces plasma homocysteine levels without altering the lipid profile both in patients with cardiovascular disease (CVD) [91] and in physically active individuals [92]. This ability to regulate vascular tone and lower homocysteine levels is highly relevant for athletes, as exercise-induced endothelial stress requires optimal blood flow and nutrient delivery. This effect is further supported by the finding that betaine administration exerts atheroprotective properties in S-adenosylhomocysteine hydrolase-deficient heterozygous knockout mice, as evidenced by a significant reduction in plasma S-adenosylhomocysteine levels [93]. Although these transgenic models strictly serve to elucidate pathways activated under pathological stress, they nevertheless validate the protective capacity of betaine. On the other hand, individuals with insufficient dietary betaine intake and concomitant homocysteine accumulation exhibit elevated plasma cholesterol concentrations and an increased risk of CVD [10].

Consequently, the vasodilatory and sport injury rehabilitation properties of betaine is enhanced when administered within beetroot juice or powder. This synergism may be driven by other naturally occurring components, such as NO_3_, and is likely mediated by an “entourage effect”—a phenomenon extensively documented in cannabis research [94,95] and also proposed for honey [96,97,98]. Analogous to the “entourage effect,” it has been demonstrated that beetroot juice consumption can yield superior ergogenic and physiological effects compared to the administration of betaine alone. This phenomenon has been described as a phytochemical synergy inherent to the whole-food matrix, in which betaine coexists with inorganic nitrates, betalains, vitamin C, and polyphenols [99,100]. Emerging evidence suggests that these co-occurring bioactive components simultaneously enhance biological pathways. For instance, polyphenols and vitamin C have been shown to optimize NO bioavailability, thereby amplifying the vasodilatory response initiated by betaine in athletes following beetroot ingestion [101]. Due to this synergistic interplay, findings obtained from beetroot juice or powder supplementation should be approached with caution when isolating the specific efficacy of betaine, necessitating a more contextualized interpretation.

### 5.5. Neuroprotective Effect

The neuroprotective effect of betaine is of particular relevance in sports, not only regarding head injuries encountered in contact sports, but also in other demanding situations. Given the high incidence of sport-related traumatic injuries, repetitive head impacts, sport-related concussion and subconcussion impacts, evaluating the neuroprotective role of betaine represents a crucial line of inquiry.

The main mechanism is the removal of homocysteine, a neurotoxic compound associated with ischemic stroke and neuronal death [102,103]. Homocysteine can act as an agonist at the glutamate binding site of N-methyl-D-aspartate (NMDA) receptors [103,104], particularly the GluN2B-NMDA subtype [103,105]. Chronic activation of these receptors by homocysteine leads to neurotoxicity and neuronal death through the phosphorylation of extracellular-signal-regulated kinase (ERK) and mitogen-activated protein kinase (MAPK) [105]. At the cerebral level, homocysteine triggers microglial activation leading to cognitive impairment [106]. Betaine supplementation in Sprague Dawley rats not only attenuates ROS, IL-18, IL-1β production [106,107], but also suppresses microglial pyroptosis by downregulating the expression of NLRP3, procaspase 1, gasdermin D (GSDMD), and GSDMD N-terminal fragment [106]. This provides neuroprotection in both dementia [90] and ischemia/reperfusion injury [107].

In preclinical models of traumatic brain injury, betaine administration has been shown to attenuate brain damage by decreasing caspase-3 and cytochrome c activity [107]. Based on mechanistic findings, it has been hypothesized that betaine supplementation could offer neuroprotective benefits in competitive athletes. However, direct clinical evidences demonstrating a reduction in brain concussion risk or specific protective efficacy, particularly in young women, remains limited and warrants further human investigations [108]. Similarly, in cerebral hemorrhage, betaine has the potential to inhibit ferroptosis by upregulating nuclear factor erythroid 2-related factor 2 (Nrf2), heme oxygenase-1, while increasing reduced glutathione levels and decreasing malondialdehyde [109].

Anxiety and depression can occur in the context of injuries, as well as due to the marked psychological pressure surrounding major sports competitions and daily training. Psychological stress, anxiety and depression represent significant changes in both elite and community sports, often triggered by performance anxiety and cumulative exposure to chronic stress [110]. This negatively influences athletes’ mental energy, which plays an essential role in both training and competitions [111]. Prolonged psychological distress, especially in elite athletes, may activate the hypothalamic–pituitary–adrenal axis, leading to elevated cortisol levels [112]. Moreover, acute competitive stress may trigger a cascade of neuroinflammatory events, where mitochondrial distress and DNA micro-damage in the hippocampus activate the proinflammatory cyclic GMP-AMP synthase-stimulator of the interferon gene (cGAS-STING) pathway, suppressing neurogenesis and exacerbating cognitive fatigue. Betaine supplementation serves as a neurological buffer by directly inhibiting cGAS-STING signaling. In this way, betaine preserves executive cognitive function under intense pressure [113]. Other research demonstrated that betaine optimizes the GABA-ergic system by downregulating expression of GABA transaminase, increasing the available GABA at the level of synapses and being responsible for the calming effect [5].

Another area in sports where betaine can intervene is alleviating depressive behavior, which can occur at some point in the life of any high-performance athlete due to overtraining, chronic physical stress, and competitive pressure. While clinical studies regarding the implications of betaine in athlete depression are still lacking, preclinical data provide valuable insights suggesting its possible antidepressant potential. In ICR mice, it has been shown that betaine-induced reduction in NLRP3 inflammasome expression induced by betaine decreases lipopolysaccharide-induced depressive behavior [114]. Betaine’s involvement in this anti-inflammatory pathway may be highly relevant to sports medicine, as elite athletes frequently exhibit elevated levels of pro-inflammatory cytokines during competitive periods and phases of intense training. Additionally, in C57BL/6 mice, pain-associated depressive behavior induced by complete Freund’s adjuvant (CFA) was eliminating following the administration of 600 mg/kg body weight betaine [115]. This correlation between pain mitigation and mood improvement is of particular value in cases of chronic musculoskeletal pain resulting from injuries—a condition that acts as a key trigger for depressive symptoms in elite athletes. Betaine appears to not only remove homocysteine from NMDA receptors [103,104,105], but also to exert a modulatory effect of them [116]. Thus, depressive-like behavior induced by the chronic administration of ketamine, an NMDA receptor antagonist, was reduced following the co-administration of 100 mg/kg body-weight betaine in ICR mice [116]. In this combination, betaine may act as a partial agonist at the glycine-binding site of NMDA receptors. Furthermore, betaine supplementation may enhance glutamate release while increasing the expression and synaptic insertion of α-amino-3-hydroxy-5-methyl-4-isoxazolepropionic acid (AMPA) receptors [117]. However, several limitations must be considered regarding the applicability of these preclinical findings to athletes. First, the doses administered in animal models (e.g., 600 mg/kg body weight) are substantially higher than typical human dosages. Second, depression and burnout in high-performance athletes constitute a complex syndrome with psychosocial dimension that cannot be fully replicated in laboratory animals. Although these preclinical results support the theoretical rationale, future clinical trials are strictly required to validate whether betaine supplementation can effectively produce these effects in high-performance athletes.

Although a number of other studies support the cognitive benefits of betaine and beetroot juice consumption [118,119,120,121], other investigations on human subjects fail to identify this benefit [122,123]. This discrepancy may be attributed to the specific demographic studied, such as obese and elderly individuals [122], combined with the low compliance observed in this category [123]. While preclinical models suggest significant neuroprotective potential, direct evidence demonstrating a role for betaine in preventing concussions or mitigating depression in athletes remain limited. Animal findings reveal promising outcomes for future research, but they cannot be yet extrapolated to clinical effects in human populations.

## 6. Discussions and Future Perspectives

The contradictory results of betaine use across different studies represent one of the most highly debated topics in sports science. The following variables should be taken into consideration. The first issue is raised by the training status of the subjects. Trained individuals have already developed homeostatic adaptation mechanisms to physical exertion, allowing betaine to effectively contribute during bouts of intense exhaustion. Conversely, in untrained individuals, the physiological response to exercise can be so overwhelming that the ergogenic effect of betaine becomes insignificant.

Another critical factor is the environment in which the study is conducted, alongside the hydration of the subjects. If the exercise session is of short duration, performed in a well-ventilated training facility with fully hydrated athletes, the osmolytic effect of betaine becomes redundant. On the other hand, during prolonged physical exertion carried out outdoors, in summer conditions, where dehydration becomes a significant factor, the ergogenic effect of betaine is distinctly noticeable.

The modality of physical exertion under evaluation should be considered. Betaine exhibits a more pronounced effect under anaerobic rather than aerobic conditions. Furthermore, in the case of prolonged, high-intensity intermittent exercise characterized by frequent changes in pace, betaine is likely to produce measurable performance improvements.

Genetic variability and dietary habits may contribute to the abundance of methyl donors available in the body. If these methyl donors are already present in high concentrations within the athlete’s body, either through exogenous dietary intake or individual enzymatic variables, the ergogenic effect of betaine will be reduced or insignificant.

The adulteration of dietary supplements, especially when complex mixtures containing multiple classes of compounds are used, is a hypothesis that must be taken into account. To eliminate this variable, it would be ideal to perform an LC-MS analysis before the experimental procedure rather than relying solely on the composition stated on the product label.

Another critical aspect to consider involves in the regulations governing the use of betaine dietary supplements. According to the European Food Safety Authority’s Novel Food Regulations, betaine is classified as a free-access dietary supplement, and safety evaluations have examined specific intake levels of 2.5 g/day for individuals over 10 years old engaging in intense physical exertion [124,125]. While standard ergogenic intakes range from 1.25 to 5 g/day are widely studied and well tolerated by elite athletes [14,19], potential adverse effects associated with high consumption among the general population must be noted [18]. The most frequently reported adverse effects include digestive discomfort, manifesting as transient diarrhea [126]. Additionally, mild elevations in total cholesterol and LDL-cholesterol levels have been documented at higher intakes exceeding 4 g/day [18]. Furthermore, betaine poses no anti-doping or toxicological risks, as its natural and synthetic sources do not contain any compounds prohibited by World Anti-Doping Agency (WADA) [127].

The transition toward precision sports nutrition and the evolution of molecular biology open up new perspectives regarding the use of this compound in peak athletic performance. Consequently, several promising directions warrant deep exploration in the future. Synergistic co-supplementation and sequential formulations represents a challenge. Instead of contrasting betaine with creatine or other ergogenic substance, future perspectives should focus on personalized, synergistic co-supplementation protocols. These interventions must be tailored not only to the athlete’s individual physiological profile and physical characteristics, but also to the specific demands of their sports, particularly regarding alternating aerobic–anaerobic energy systems.

The utilization of betaine may be considered for its cardiovascular and neuroprotective benefits. Prolonged physical exertion and competitive pressure induce severe physiological stress leading to an increase in inflammatory markers and homocysteine levels. Consequently, isolated betaine supplementation, or a diet rich in natural sources such as beetroot, could establish this compound as a vital agent for maintaining homeostatic balance and promoting athletic longevity.

Epigenetic optimization through DNA methylation capacity represents a promising avenue with substantial development potential. Future research should explore this mechanism, which is currently investigated primarily in animal models [128,129,130,131,132]; however, these findings are beginning to be translated into human studies involving cohorts with CVD [133]. Ultimately, applying this epigenetic framework to sports nutrition could allow athletes to maximize their adaptive response to training stimuli.

Biomarker-based nutritional personalization holds significant potential for integration in the future framework of personalized medicine. This approach involves tailoring nutritional guidance to the athlete’s genetic profile, particularly regarding the MTHFR genotype, which may respond more effectively to targeted betaine supplementation [134].

The future prospects of betaine as an ergogenic aid depend heavily on independent homogeneous studies involving elite athletes. Such research is essential to clarify current controversies surrounding isolated betaine supplementation, its multi-ingredient combinations and the “food-first” approach through the exploration of natural food sources.

## 7. Conclusions

The utilization of betaine anhydrous in sport nutrition has been characterized by a unidirectional paradigm, primarily focusing on its dual function as an osmolyte and a methyl donor. Consequently, research and practical applications have focused on its capacity to mitigate cellular stress during high-intensity muscular exertion and to optimize time-to-exhaustion thresholds. However, the post-injury recovery capacity of betaine could be beneficial in facilitating the metabolic clearance of homocysteine, a potentially dangerous metabolite. Within athletes, its anti-inflammatory, adipomodulatory and neuroprotective properties merit careful consideration, concurrently with its possible capacity to augment NO bioavailability and optimize ergogenic outcomes in humans. Although these physiological contributions may appear marginal initially, such minor advantages are magnified during elite-level competitions. Ultimately, these incremental enhancements can dictate the boundary securing a podium finish or experiencing an exclusion from a higher competitive hierarchy.

## Figures and Tables

**Figure 1:**
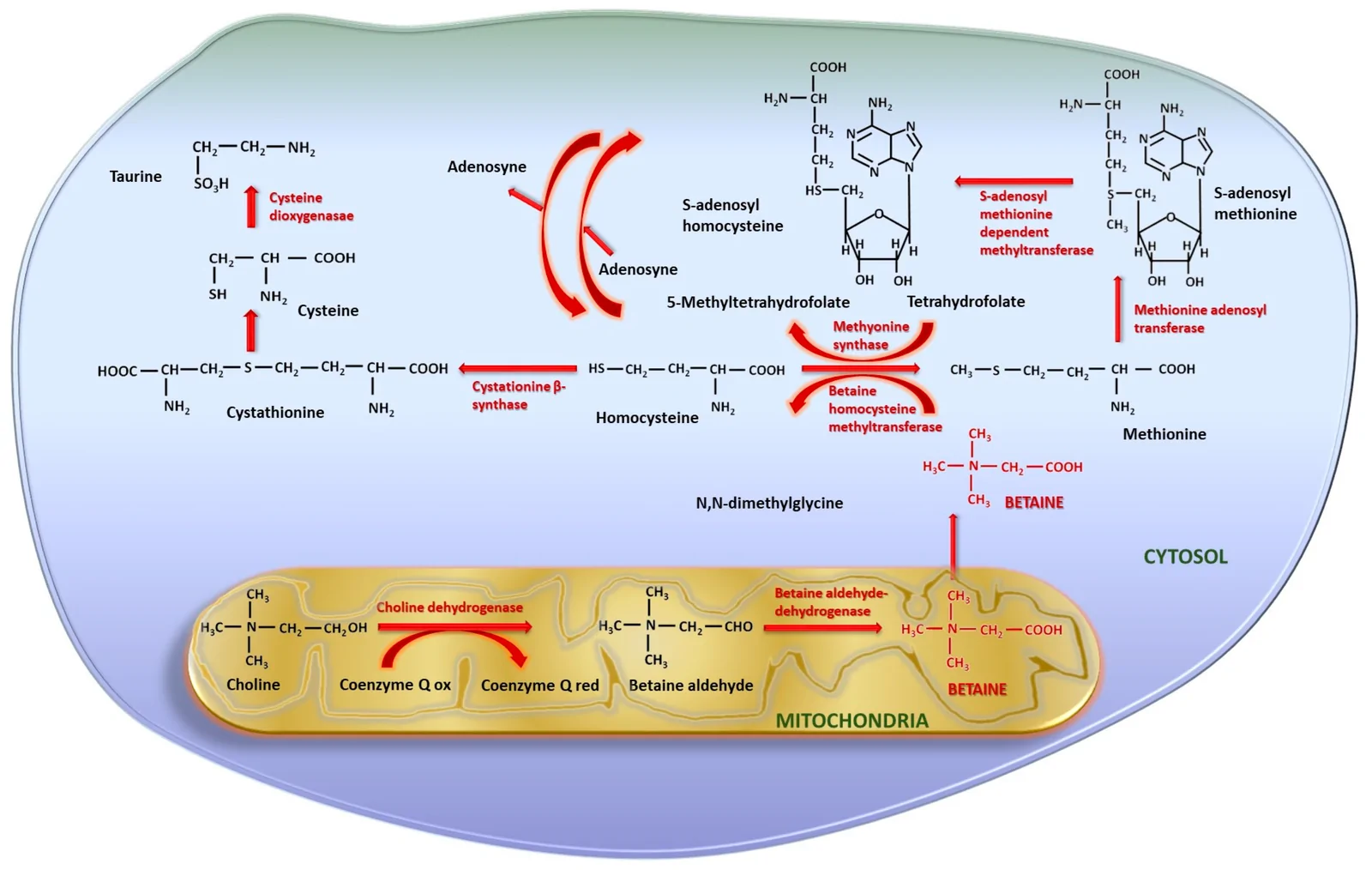
Schematic representation of betaine synthesis and its related metabolic transformations.

**Figure 2:**
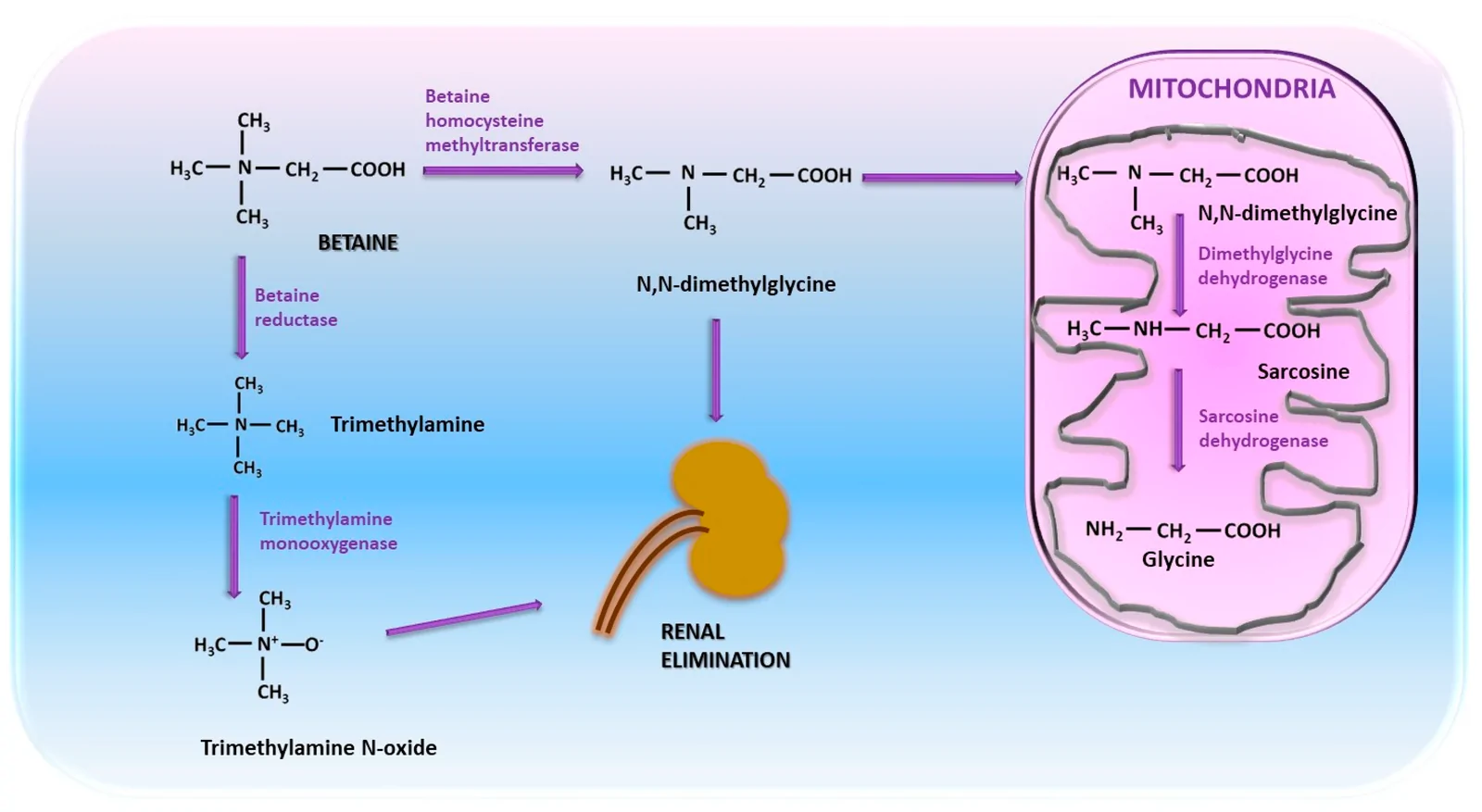
Metabolic pathways of betaine and the elimination of its catabolic products.

**Figure 3:**
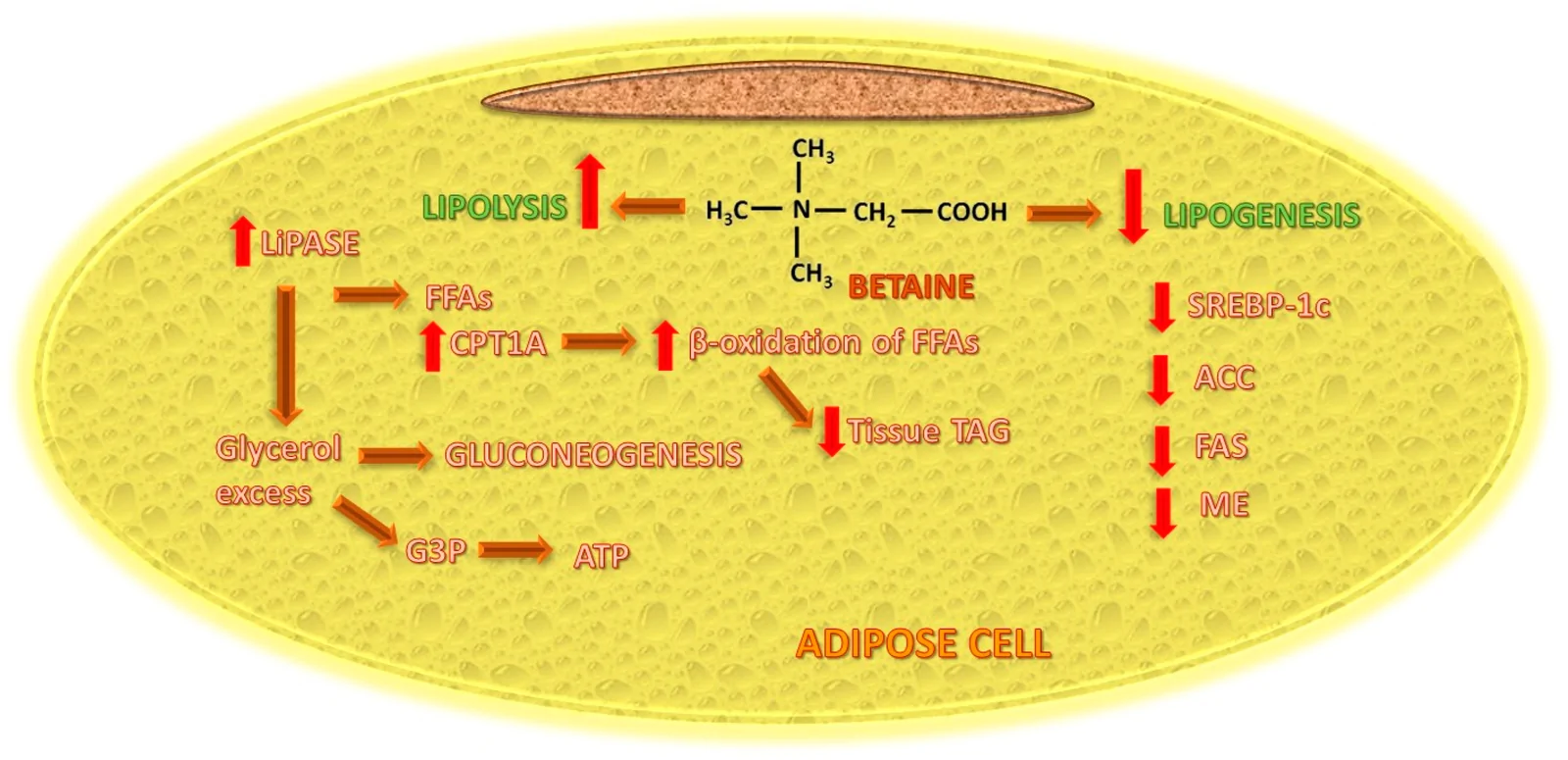
The anti-adipogenic effect of betaine and its impact on lipolysis and lipogenesis. FFAs—free fatty acids; SREBP-1c—sterol regulatory element-binding protein-1c; CPT1A—carnitine palmitoyl transferase 1A; TAG—triacylglycerol; ACC—acetyl-CoA carboxylase; FAS—fatty acid synthase; ME—malic enzyme; G3P—glycerol 3-phosphate.

**Table 1:** Randomized controlled trials evaluating the impact of betaine supplementation and athletic performance outcomes.

Study Type	Population/Subjects	Dose and Intervention	Duration	Outcome	Reference
1. Randomized, double-blind	Trained CrossFit male and female athletes	2.5 g/day of betaine supplementations versus placebo in combination with training 2/3 times a week	6 weeks	Betaine supplementation increased submaximal strength, but no results were reported in body composition or strength endurance.	[20]
2. Double-blind, crossover study design	Young handball players	2.5 g/day of betaine compared with maltodextrin placebo	14-day intervention with a 30-day washout period	Muscular endurance was improved, along with an increased testosterone level and lowered post-exercise cortisol and lactate.	[53]
3. Randomized, placebo-controlled	Healthy female and male cyclists, capable of cycling 60 km	3 g/day of betaine versus placebo with a 60 km cycling time trial after each supplementation	2 weeks of betaine supplementation versus 2 weeks of placebo	Betaine significantly reduced the time required to complete a 60 km cycling race, without influencing gut permeability, muscle damage, inflammation, or cortisol levels.	[7]
4. Randomized, double-blind, placebo-controlled mixed-model	Men in good health, recreationally resistance-training, aged 18–35 years	6 g total per day of betaine anhydrous versus cellulose placebo, followed by blood flow restriction (BFR) training	14 days of supplementation	Betaine increased IGF-1 response, but no clear BFR–betaine synergistic effect was detected.	[54]
5. Randomized, double-blind, placebo-controlled	Untrained young women	2.5 g/day of betaine supplementation followed by resistance training versus placebo and resistance training	8 weeks	Good results were obtained in reducing body fat, but there were no notable results in terms of strength, muscle thickness, or vertical jumping.	[55]

## Data Availability

No new data were created or analyzed in this study. Data sharing is not applicable to this article.

## Abbreviations

The following abbreviations are used in this manuscript:

ACC	acetyl-CoA carboxylase
AMPA	α-amino-3-hydroxy-5-methyl-4-isoxazole propionic acid
AMPK	AMP-activated protein kinase
CFA	complete Freund’s adjuvant
cGAS-STING	proinflammatory cyclic GMP-AMP synthasestimulator of interferon genes
CPT1A	carnitine palmitoyl transferase 1A
CVD	cardiovascular diseases
eNOS	endothelial nitric oxide synthase
ERK	extracellular-signal-regulated kinase
FFA	free fatty acid
GH	growth hormone
GSDMD	gasdermin D
HFD	high fat diet
HIF	hypoxia-inducible factor
IGF-1	insulin-like growth factor-1
MAPK	mitogen-activated protein kinase
ME	malic enzyme
MMP	matrix metalloproteinase
NF-kB	nuclear factor-kB
NLRP3	leucine-rich pyrin-containing-3
NMDA	N-methyl-D-aspartate
Nrf2	erythroid 2-related factor 2
PGC1α	peroxisome proliferator-activated receptor γ coactivator 1α
ROS	reactive oxygen species
SHR	spontaneously hypertensive rats
SREBP-1c	sterol regulatory element-binding protein-1c
TAG	triacylglycerol
TLR4	toll-like receptor 4
TMAO	trimethylamine N-oxide
WADA	World Anti-Doping Agency
WKR	Wistar Kyoto rat
